# Supplementary material for: 5-Oxo-ETE/OXER1: A Link between Tumor Cells and Macrophages Leading to Regulation of Migration
Source: Molecules. 2023 Dec 31;29(1):224. doi: 10.3390/molecules29010224 (PMC10780139; doi:10.3390/molecules29010224)
Supplement: Supplementary file 1 [file molecules-29-00224-s001.zip › molecules-2744504-supplementary.pdf]

5-oxo-ETE/OXER1 a link between tumor cells and macrophages leading to regulation of migration

Kalyvianaki K[1], Salampasi EM[1], Katsoulieris EN[1], Boukla E[1], Vogiatzoglou AP[1], Notas G[1], Castanas E[1], Kampa M[1]

SUPPLEMENTAL FIGURES

Supplemental Figure 1

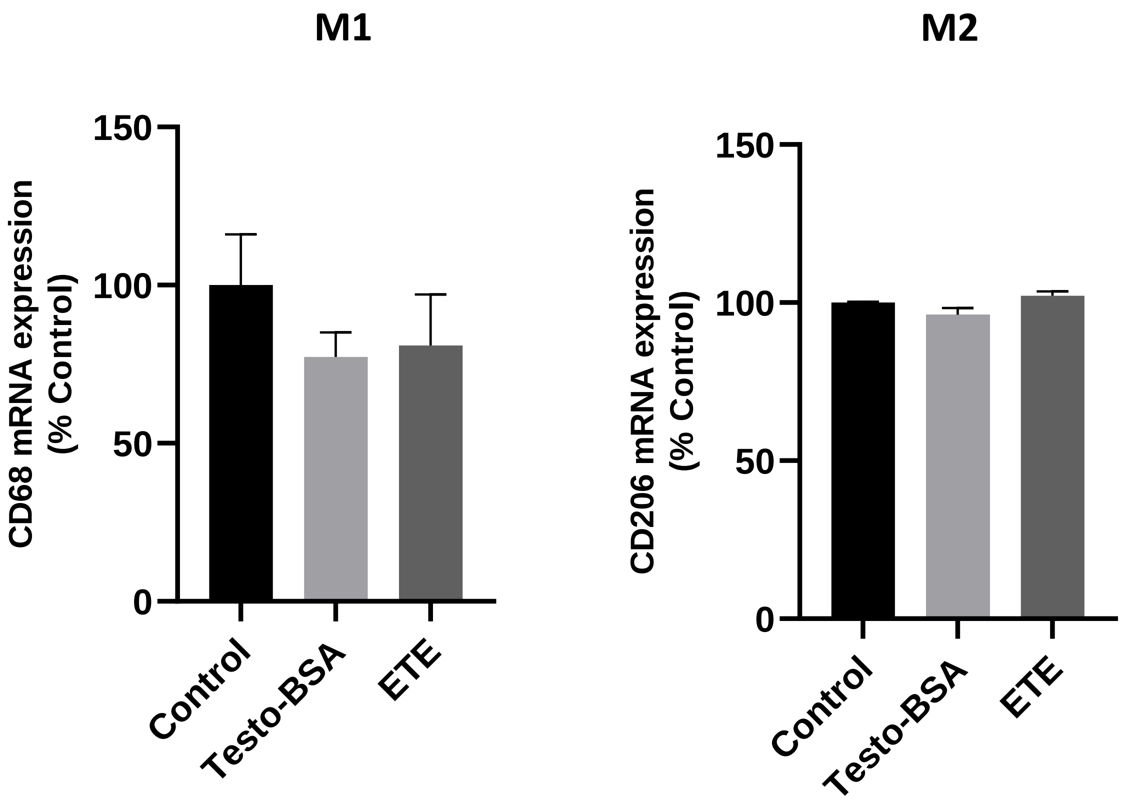

5-oxo-ETE and testosterone-BSA effect on CD68 and CD206 mRNA levels in M1 and M2 macrophages respectively. THP-1 PMA treated cells were treated for 6 hours before the addition of IFNγ/LPS or IL-4.

Supplemental Figure 2

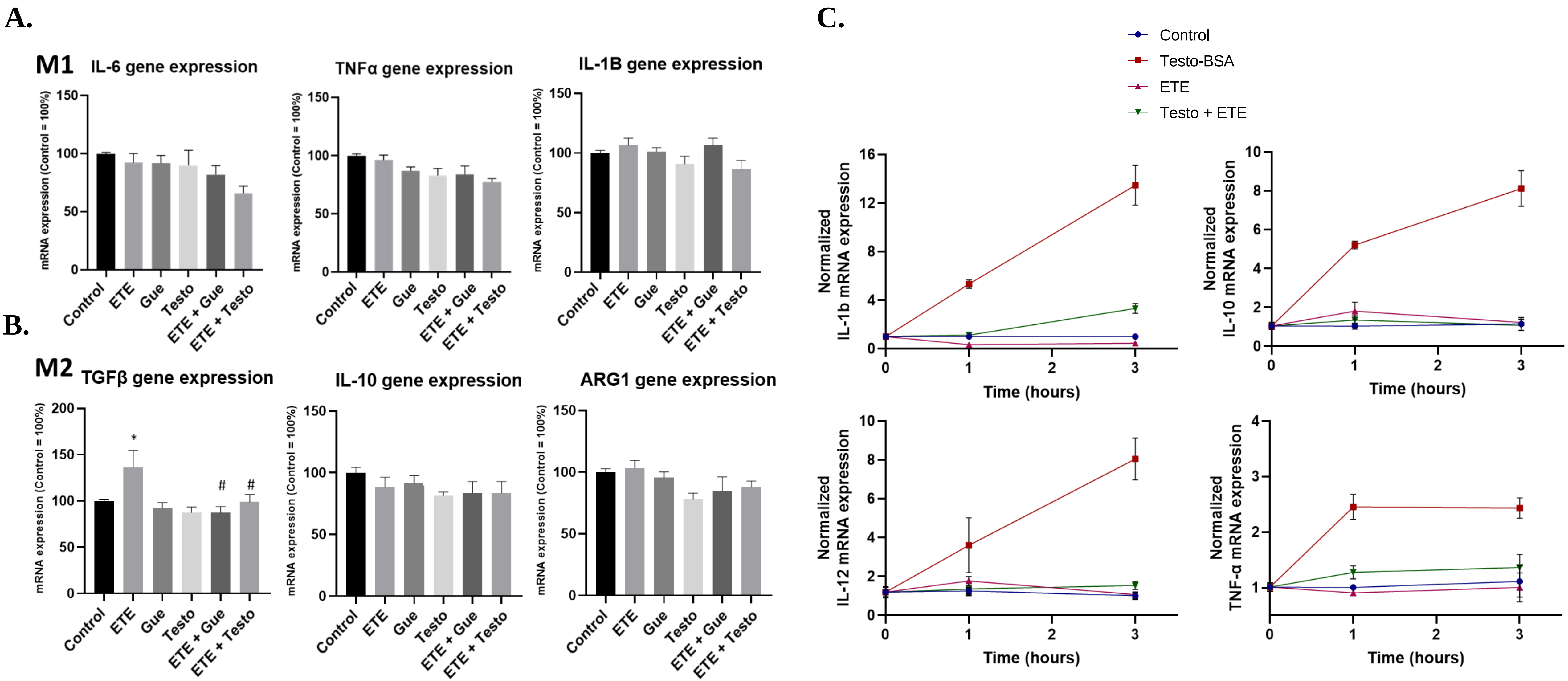

OXER1 expression in inflammatory cells and the effect of the receptor's ligands in macrophages' cytokines. Cytokines expression was measured with qRT-PCR after 1 or 3h treatment with 5-oxo-ETE (10−6 M) and its antagonists in M1 (A), M2 (B) and THP-1 (C) cells. THP-1 and THP-1 differentiated cells express different levels of OXER1. Results are shown as Mean ± SE of 3 independent experiments. Statistical significance vs control * $p<0.05$, vs 5-oxo-ETE # $p<0.05$

Supplemental Figure 3

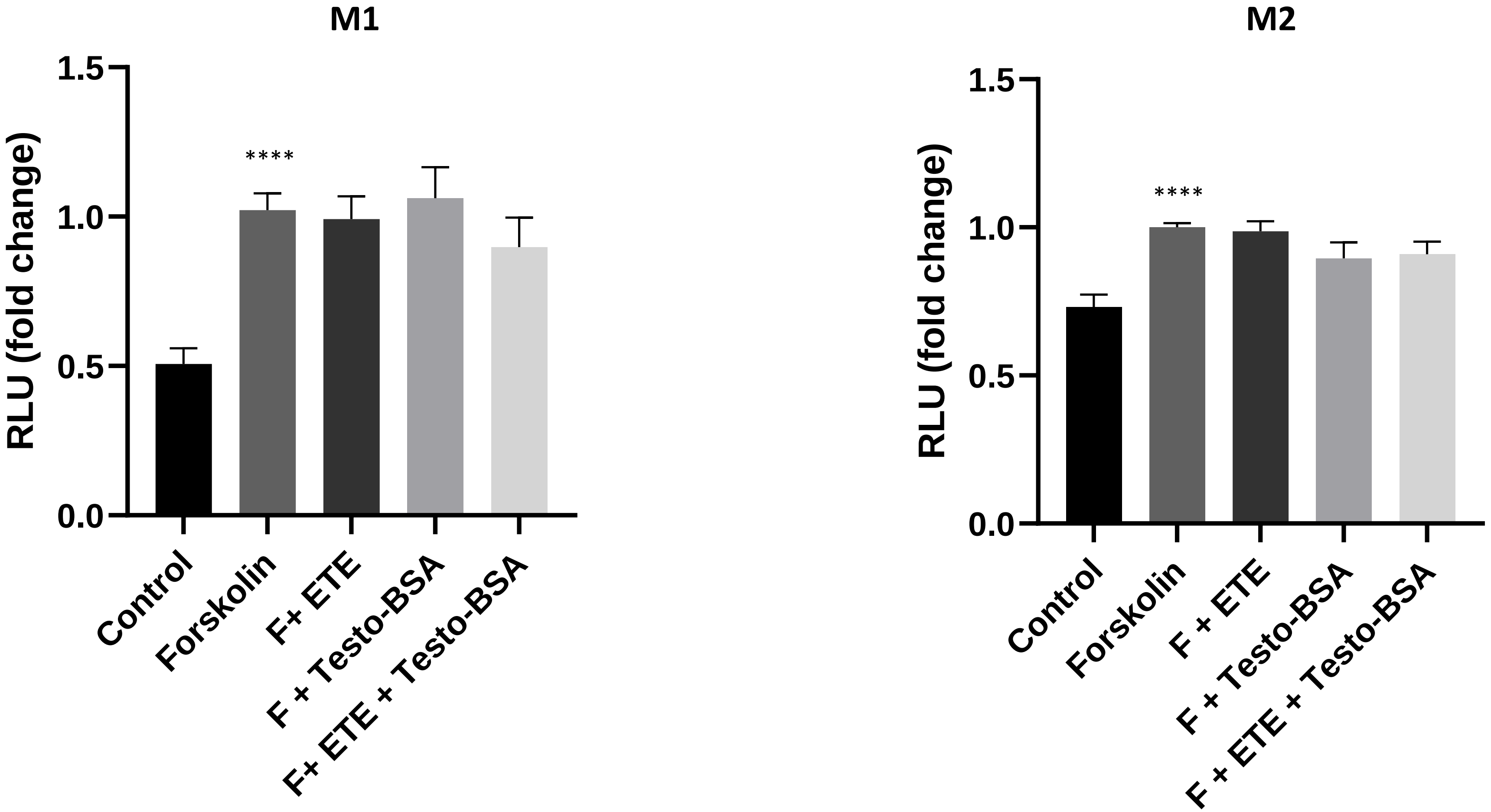

cAMP regulation by Forskolin in the presence or absence of OXER1 ligands in M1 and M2 macrophages. Results are shown as Mean ± SE of 3 independent experiments. Statistical significance vs control **** $p<0.0001$

Supplemental Figure 4

DU-145

T47D

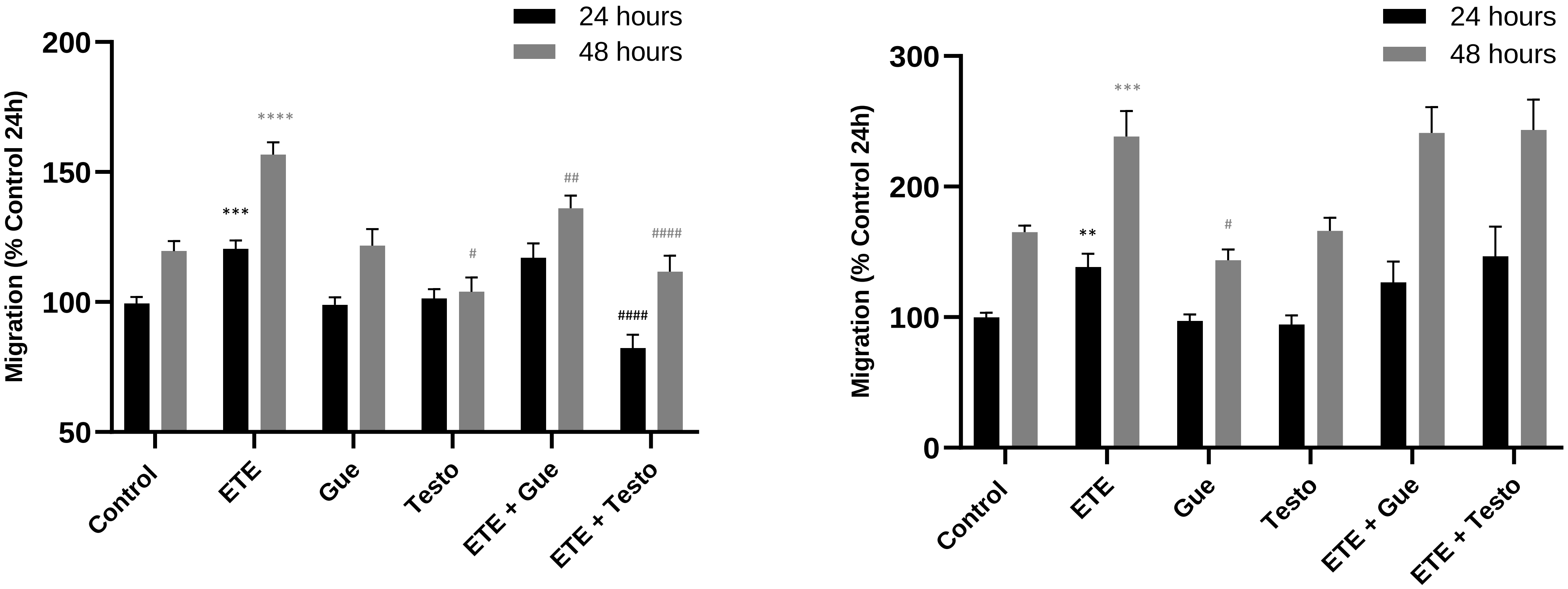

Migration of DU-145 and T47D cells induced by 5-oxo-ETE. Normalized wound healing of DU-145 and T47D cells treated with 5-oxo-ETE, testosterone-BSA, Gue 1654, their combination (all treatments at 10^{-6} M) or the appropriate vehicle (control). The wound progression was observed for each cell line at 0h, 24h and 48h. Results are shown as Mean ± SE of 3 different experiments. Statistical significance vs control * $p<0.05$, ** $p<0.01$, *** $p< 0.001$, **** $p<0.0001$, vs 5-oxo-ETE # $p<0.05$, ## $p<0.01$, ### $p<0.001$, #### $p<0.0001$

Supplemental Figure 5

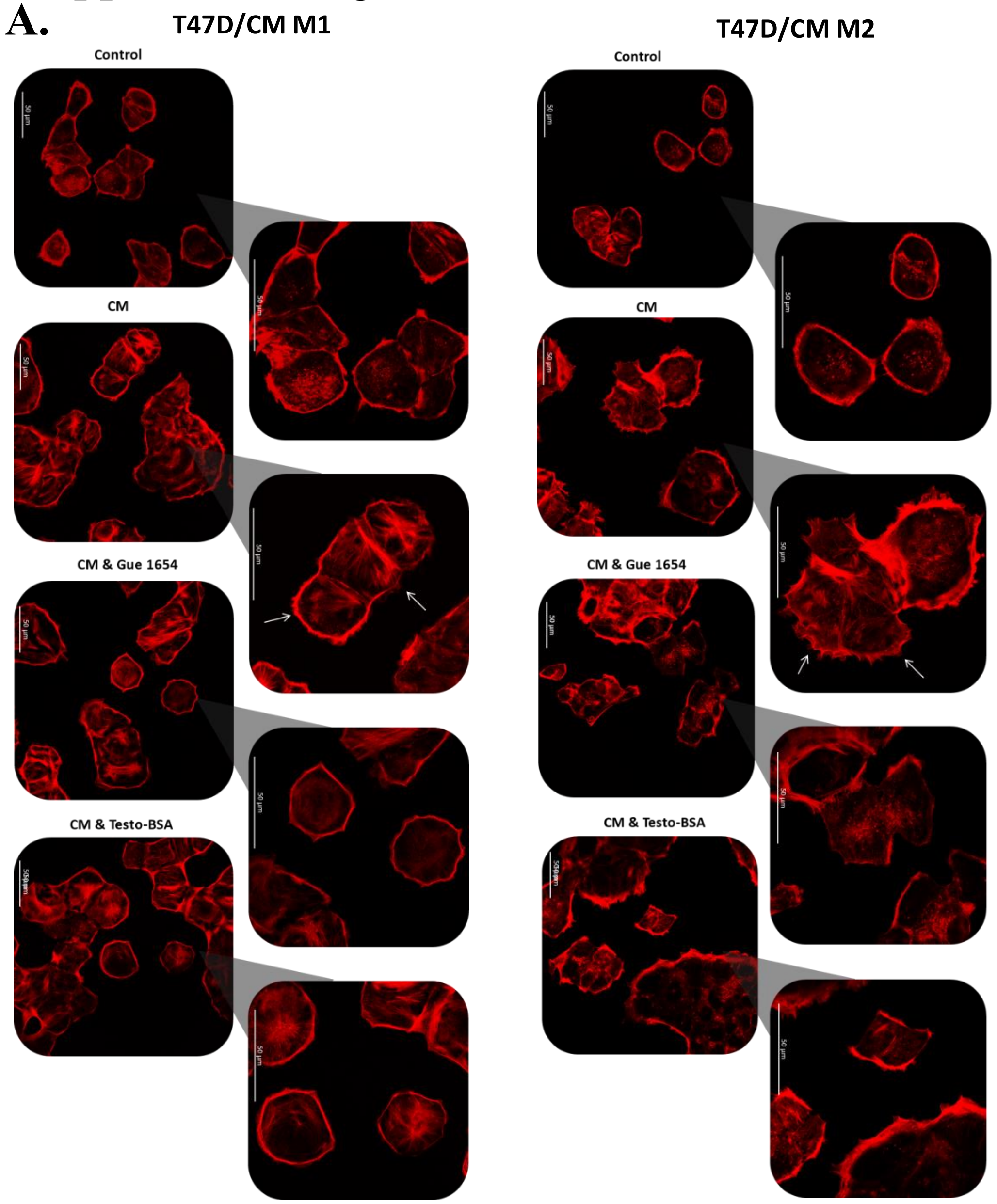

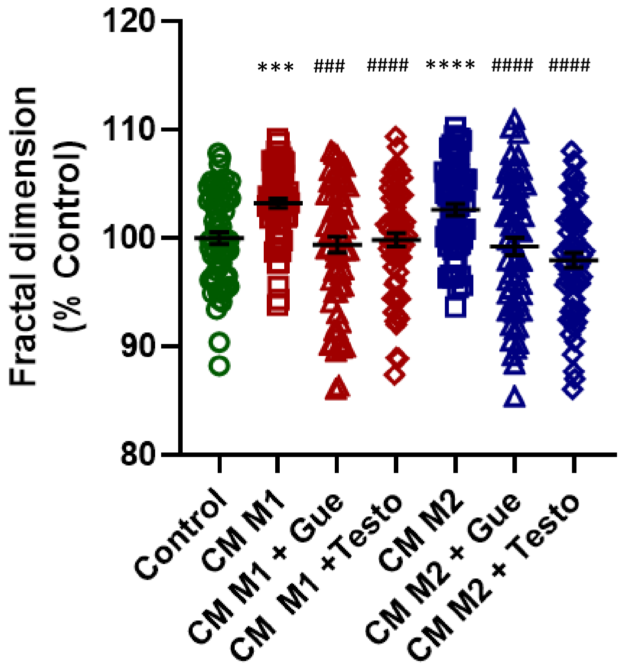

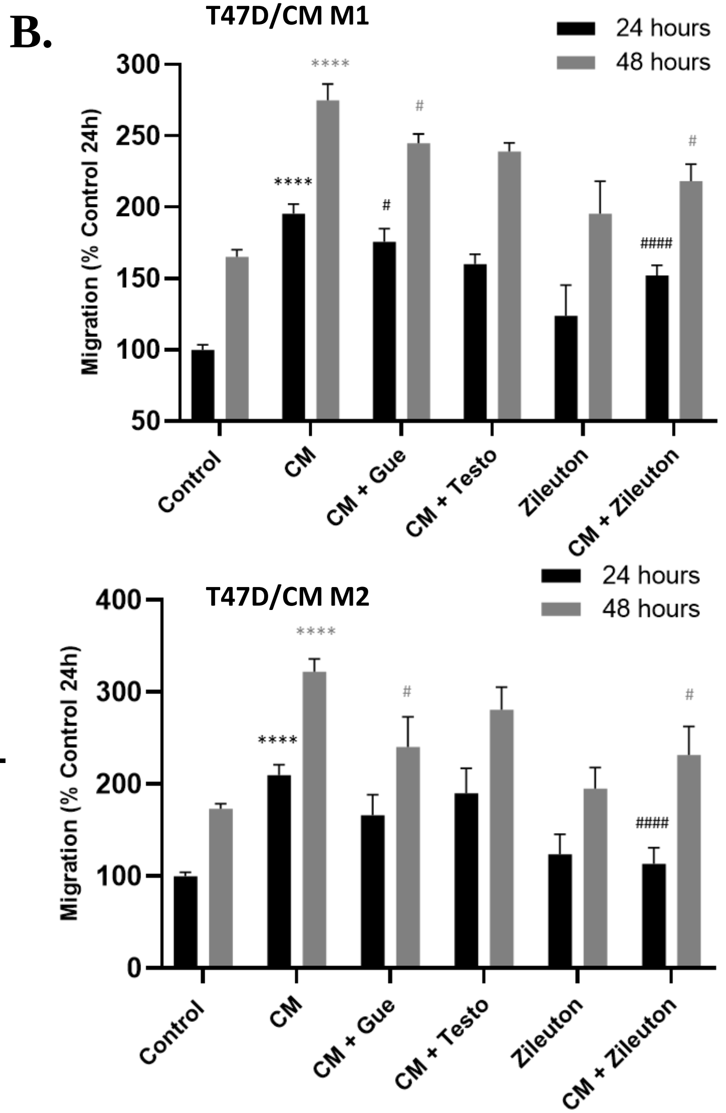

Redistribution of actin filaments (A) and migration (B) of T47D after treatment with conditioned medium (CM) from M1 or M2 macrophages. T47D were treated with CM in the presence or absence of testosterone-BSA, Gue 1654 and Zileuton (all at 10^{-6} M) for 30 min and **A.** the redistribution of filamentous actin was assessed using rhodamine-phalloidin. Representative photos of three independent experiments are shown. White arrows indicate the formation of filopodia. Fractal dimension analysis of 60 cells per treatment. Results are shown as Mean ± SE. Statistical significance vs control **** $p<0.0001$, vs CM # $p<0.05$, ### $p<0.001$, #### $p<0.0001$. **B**. Migration of T47D after 24 and 48h. Results are shown as Mean ± SE of three independent experiments. Statistical significance vs control * $p<0.05$, *** $p<0.001$, **** $p<0.0001$, vs CM # $p<0.05$, ## $p<0.01$, ### $p<0.001$, #### $p<0.0001$.

Supplemental Figure 6

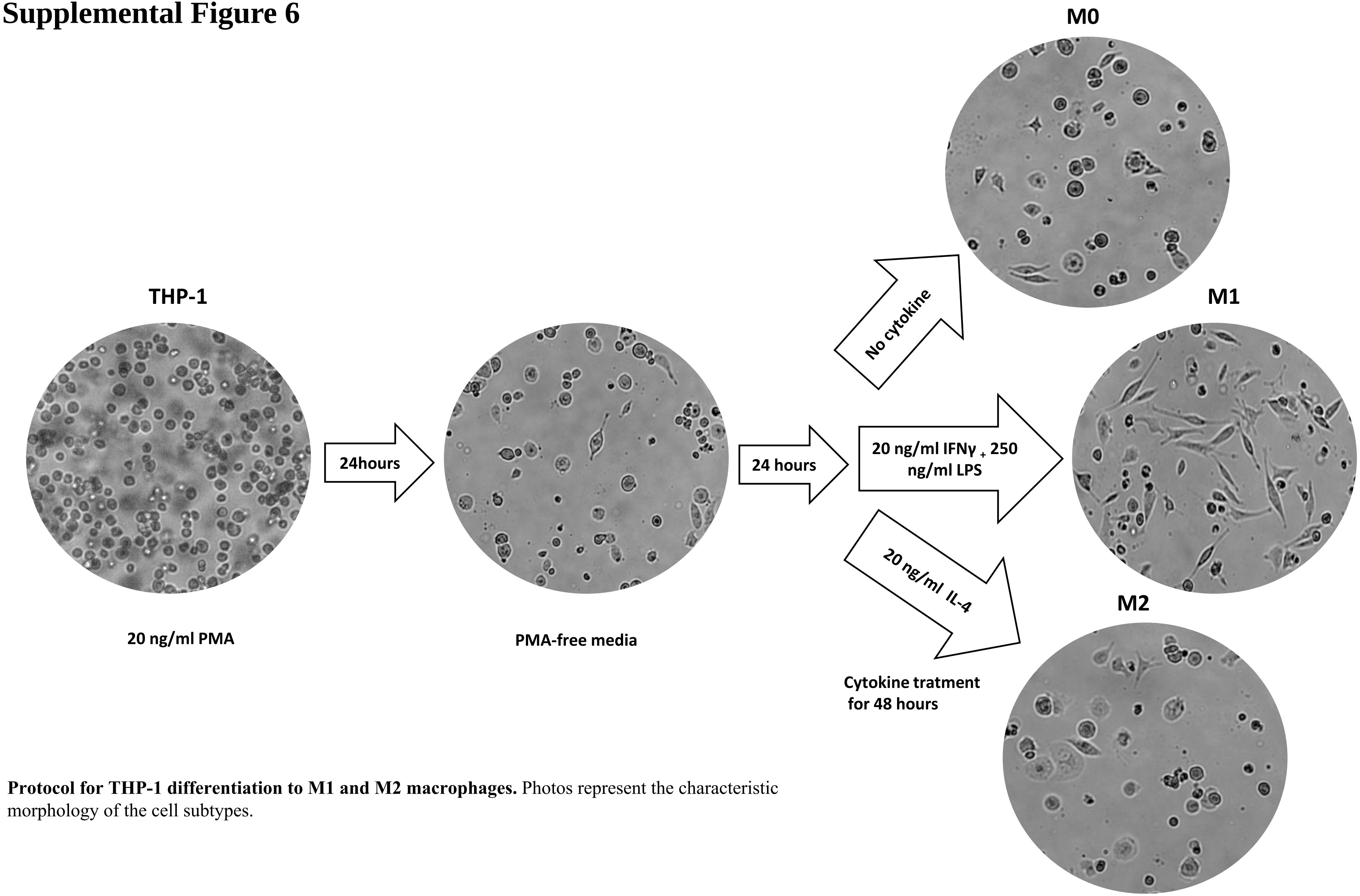

Protocol for THP-1 differentiation to M1 and M2 macrophages. Photos represent the characteristic morphology of the cell subtypes.

Supplemental Figure 7

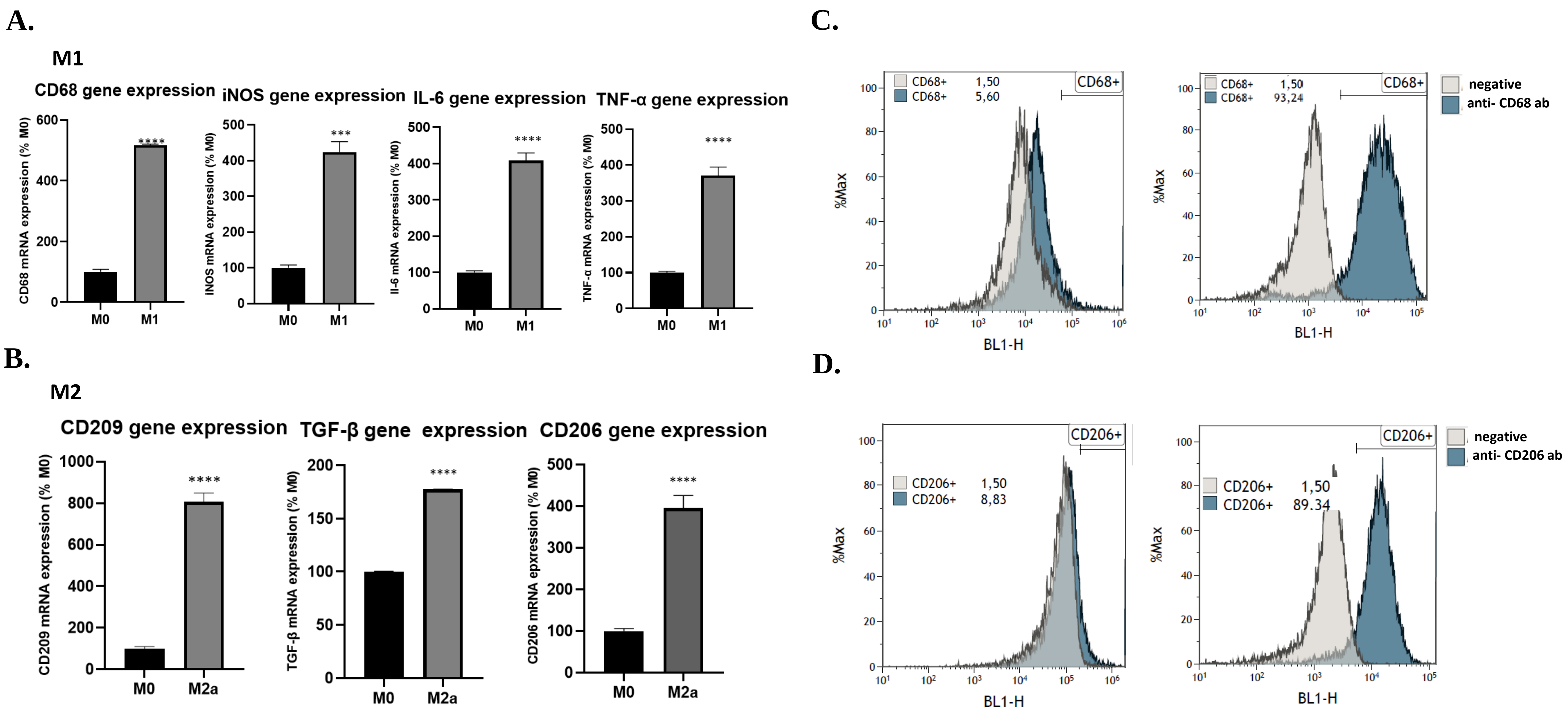

Verification of THP-1 differentiation. Specific CD markers and cytokines were used for M1 (A) and M2 (B) in order to verify the macrophage-like characteristics of these cells after THP-1 differentiation. M1 (C) and M2 (D) cells were stained with CD68 and CD206 abs and the protein expression of the retrospective genes was assayed by FACS. Verification assays were performed after each differentiation and before subjecting the cells to another assay. Results are shown as Mean ± SE (A, B) Statistical significance vs M0 *** $p<0.001$, **** $p<0.0001$

SUPPLEMENTAL TABLES

SUPPLEMENTAL TABLE 1

**PROTEIN**	**ANTIBODY**	**DILUTION**
CD45	CD45 PerCP Clone MEM-28, EXBIO	1:200
CD68	Invitrogen, eBioscienceTM, 14-0688-82	1:100
CD206	R&D SYSTEMS, biotechne, MAB25341	1:200
OXER1	PA5-75410, Invitrogen	1:100
Mouse anti-rabbit IgG-CFL 555	Santa Cruz Biotechnology	1:500
Alexa Fluor 488	Invitrogen Molecular Probes	1:500
CD3 - FITC	BD Pharmingen (BD biosciences) (555332)	1:50
CD4 - PE	BD Pharmingen (BD biosciences) (555347)	1:50
CD8 - PE	BD Pharmingen (BD biosciences) (555635)	1:50
CD19 - FITC	BD Pharmingen (BD biosciences) (555412)	1:50
CD20 - PE	BD Pharmingen (BD biosciences) (555623)	1:50
CD14 - FITC	BD Pharmingen (BD biosciences) (555397)	1:50
CD11b - PE	BD Pharmingen (BD biosciences) (333142)	1:50
CD16 - PE	BD Pharmingen (BD biosciences) (555407)	1:50

SUPPLENENTAL TABLE 2

**GENE**	**Forward Primer (5'→3')**	**Reverse Primer (3'→5')**
**OXER1**	AGGAGCCTTCCTTTTCCAGA	CGACGAGAGCTCCTACCAAC
**CD68**	CGAGCATCATTCTTTCACCAGCT	ATGAGAGGCAGCAAGATGGACC
**IL-6**	AGACAGCCACTCACCTCTTCAG	TTCTGCCAGTGCCTCTTTGCTG
**TNF-a**	AGATGATCTGACTGCCTGGG	CTGCTGCACTTTGGAGTGAT
**IL-1b**	AATCTGTACCTGTCCTGCGTGTT	TGGGTAATTTTTGGGATCTACACTCT
**iNOS**	CTGTCCTTGGAAATTTCTGTT	TGGCCAGATGTTCCTCTATT
**CD209**	GCAGTCTTCCAGAAGTAACCGT	GCTCTCCTCTGTTCCAATACTGC
**CD206**	AGCCAACACCAGCTCCTCAAGA	CAAAACGTCGCGCATTGTCCA
**TGF-β**	CCCAGCATCTGCAAAGCTC	GTCAATGTACAGCTGCCGCA
**Arginase 1**	CTCAAAGGGACAGCCACGAG	CAAAGGGCAGGTCCCCATAA
**IL-10**	GCGCTGTCATCGATTTCTTCC	GCCACCCTGATGTCTCAGTT
**Cyclophilin A**	ATGGTCAACCCCACCGTGT	TTCTGCTGTCTTTGGAACTTTGTC
